# MicroRNA-135a Modulates Hepatitis C Virus Genome Replication through Downregulation of Host Antiviral Factors

**DOI:** 10.1007/s12250-018-0055-9

**Published:** 2018-11-19

**Authors:** Catherine Sodroski, Brianna Lowey, Laura Hertz, T. Jake Liang, Qisheng Li

**Affiliations:** Liver Diseases Branch, National Institute of Diabetes and Digestive and Kidney Diseases, National Institutes of Health, Bethesda, 20892 USA

**Keywords:** Hepatitis C virus (HCV), Genome replication, Virus–host interactions, miR-135a, Antiviral factors

## Abstract

Cellular microRNAs (miRNAs) have been shown to modulate HCV infection via directly acting on the viral genome or indirectly through targeting the virus-associated host factors. Recently we generated a comprehensive map of HCV–miRNA interactions through genome-wide miRNA functional screens and transcriptomics analyses. Many previously unappreciated cellular miRNAs were identified to be involved in HCV infection, including miR-135a, a human cancer-related miRNA. In the present study, we investigated the role of miR-135a in regulating HCV life cycle and showed that it preferentially enhances viral genome replication. Bioinformatics-based integrative analyses and subsequent functional assays revealed three antiviral host factors, including receptor interacting serine/threonine kinase 2 (RIPK2), myeloid differentiation primary response 88 (MYD88), and C-X-C motif chemokine ligand 12 (CXCL12), as *bona fide* targets of miR-135a. These genes have been shown to inhibit HCV infection at the RNA replication stage. Our data demonstrated that repression of key host restriction factors mediated the proviral effect of miR-135a on HCV propagation. In addition, miR-135a hepatic abundance is upregulated by HCV infection in both cultured hepatocytes and human liver, likely mediating a more favorable environment for viral replication and possibly contributing to HCV-induced liver malignancy. These results provide novel insights into HCV–host interactions and unveil molecular pathways linking miRNA biology to HCV pathogenesis.

**Electronic supplementary material:**

The online version of this article (10.1007/s12250-018-0055-9) contains supplementary material, which is available to authorized users.

## Introduction

Hepatitis C virus (HCV), a positive-sense, single-stranded RNA virus of the *Flaviviridae* family, chronically infects approximately 150 million people worldwide (Mohd Hanafiah *et al*. 2013). Without intervention, HCV infection often leads to progressive liver damage that induces cirrhosis, liver failure, and hepatocellular carcinoma (HCC). While highly effective direct-acting antiviral (DAA) regimens have been developed, eradication of the virus does not completely eliminate the increased risk of advanced liver diseases, including fibrosis and HCC (El-Serag *et al*. 2016). It is therefore of medical interest to elucidate the molecular mechanisms underlying HCV-associated liver dysfunctions.

HCV depends heavily on host or cellular factors to establish persistent infection and trigger unique hepatic pathological processes (Randall *et al*. 2007; Li *et al*. 2009; Tai *et al*. 2009; Lupberger *et al*. 2011; Reiss *et al*. 2011; Li *et al*. 2014). Cellular microRNAs (miRNAs) represent such host dependencies and play important roles in regulating viral infection and related pathogenesis. MicroRNAs are 22-nucleotide non-coding RNAs that repress target gene expression and manipulate a multitude of pathophysiological processes in cells. miRNAs exert their effects by binding to predominantly the 3′ untranslated regions (UTRs) of the target mRNAs, thus suppressing their expression via mRNA degradation and/or translational repression scenarios (Bartel 2009; Fabian *et al*. 2010). Host miRNAs have been shown to influence the life cycle of many viruses both directly, through interactions with the viral genome, or indirectly, through regulation of critical virus-associated cellular pathways. A prime example is the reliance of productive HCV infection on the expression of a liver-specific cellular miRNA–miR-122. Mechanistically, miR-122 binds to the HCV 5′ UTR, stimulates viral RNA synthesis and translation, and protects the viral genome from degradation by the 5′ exoribonucleases Xrn1 and Xrn2 (Jopling *et al*. 2005; Li *et al*. 2013; Sedano and Sarnow 2014; Li *et al*. 2015; Masaki *et al*. 2015). Interestingly, HCV RNA may sequester miR-122 through a “sponge” effect that causes global de-repression of cellular miR-122 targets. This mechanism potentially facilitates the long-term oncogenic features of HCV infection (Luna *et al*. 2015, 2017). HCV infection is also modulated by multiple other host miRNAs in an intricate cellular regulatory network that engages both proviral and antiviral mechanisms (Singaravelu *et al*. 2014). Dysfunction and deregulation of the cellular miRNA landscape have also been attributed to HCV-associated liver diseases, including hepatic steatosis, fibrosis and liver cancer (Szabo and Bala 2013; Bandiera *et al*. 2015).

Recently, we interrogated the entire miRNome for their impacts on HCV infection by conducting a genome-wide miRNA functional screen. Numerous interactions between cellular miRNAs and the HCV life cycle were identified (Li *et al*. 2017). Among the previously unknown HCV-associated miRNAs is miR-135a, a miRNA that is elevated broadly across many cancer types and a candidate driver of HCV-associated hepatocarcinogenesis (Van Renne *et al*. 2018). Overexpression of miR-135a mimic in cultured hepatocytes significantly enhanced HCV core protein expression and viral RNA levels, eliciting its proviral function in modulating HCV infection (Li *et al*. 2017). Nevertheless, the precise role and mode of action of miR-135a in enhancing HCV infection remain uncharacterized. Moreover, whether the expression or function of miR-135a is affected by HCV and contributes to virus-associated progressive liver damage is not yet known. In this study, we investigated the effects of miR-135a on HCV life cycle and demonstrated that this miRNA modulates HCV genome replication through its actions on several critical antiviral host factors, which have been implicated in HCV-mediated liver pathogenesis.

## Materials and Methods

### Cells and Viruses

The human hepatoma Huh7 derivative cell line Huh7.5.1 (provided by F.V. Chisari of The Scripps Research Institute, La Jolla, CA) was maintained in complete growth medium (DMEM; Corning) containing 10% Fetal Bovine Serum (Corning). The cell line was free from mycoplasma contamination, regularly tested using a Mycoplasma Detection Kit (Thermo Fisher Scientific).

HCV genotype 2a JFH-1 strain (provided by T. Wakita of the National Institute of Infectious Diseases, Tokyo, Japan) was propagated and infectivity was titrated as previously described (Wakita *et al*. 2005; Kato *et al*. 2007). The chimeric HCV cDNA clones of multiple genotypes and subtypes (provided by J. Bukh of Copenhagen University Hospital, Copenhagen, Denmark) were JFH-1–based recombinants containing the structural proteins (core, E1, and E2), p7 and NS2 of genotypes 1a (H77C), 1b (HC-J4), 2b (HC-J8), 3a (S52), and 4a (ED43), respectively. The viral stocks were prepared and titrated as described previously (Gottwein *et al*. 2009). Unless otherwise indicated, HCV infection was conducted at a multiplicity of infection (MOI) of 0.5, and assays were typically performed at 48 h post-infection.

### Patients and Liver Biopsies

Liver biopsy samples were obtained from both healthy volunteers and patients with chronic hepatitis C with genotype 1b infection. All patients provided written informed consent and the protocol was approved by the Institutional Review Board of the National Institute of Diabetes and Digestive and Kidney Diseases and the National Institute of Arthritis and Musculoskeletal and Skin Diseases.

### MicroRNA Transfection

In 12- or 96-well format, miRIDIAN human miRNA mimics or miR-122 hairpin inhibitor (Dharmacon) were transfected into Huh7.5.1 cells at a final concentration of 25 nmol/L, applying a reverse transfection protocol using Oligofectamine (Invitrogen). Typically, cells were incubated with miRNAs for 72 h, when miRNA-mediated gene regulation is robust, and then either further treated or harvested for various assays. miRIDIAN microRNA Mimic Negative Control #2 (Dharmacon) was used as the negative control in multiple assays.

### siRNA Transfection

Human ON-TARGETplus SMARTpool siRNAs (Dharmacon) were transfected into Huh7.5.1 cells at a 50 nmol/L final concentration, utilizing Oligofectamine and a reverse transfection protocol as previously described (Li *et al*. 2009). ON-TARGETplus Non-targeting siRNA #2 (siNT) was used as a negative control. Unless otherwise noted, at 72 h post-transfection, when silencing efficiency reaches its maximal level, cells were either harvested for gene expression analysis, or infected with HCV for further virological assays.

### HCV Life Cycle Assays

To evaluate the potential impacts of miR-135a on various steps of HCV infection, viral life cycle assays were conducted. Specifically, HCVpp (for entry), subgenomic replicon (for IRES-mediated translation and/or viral genome replication), HCVsc (for single cycle infection) and HCVcc (for the entire life cycle) assays were performed. Detailed assay methods are described below.

#### HCV Entry Assay

HCV pseudoparticles (HCVpp) and VSV-Gpp were generated as previously described (Li *et al*. 2014). HCVpp harboring E1/E2 glycoproteins from genotypes 1a and 1b were derived from the plasmids pHCV7a and pHCV-E1E2.1b3 (provided by F. L. Cosset of the INSERM U412, Lyon, France), respectively, as described previously (Lavillette *et al*. 2005). Huh7.5.1 cells were seeded at the density of 4500 cells per well in 96-well white microplates (Greiner Bio-one), and then transfected with various miRNA mimics (at 25 nmol/L) or SMARTpool siRNAs (at 50 nmol/L) using a reverse transfection protocol (in 5 replicates). After 72 h, cells were infected with HCVpp or VSV-Gpp. At 48 h post-infection, cells were lysed in 1 × Reporter Lysis Buffer (Promega). Firefly luciferase activity was subsequently measured per manufacturer’s instructions (Promega) using a POLARstar Omega multidetection microplate reader (BMG Labtech).

#### HCV Subgenomic Replicon Assay

Huh7.5.1 cells were treated with the mimic control, miR-135a or miR-122 mimic (at 25 nmol/L) for 72 h in 96-well white plates (in 5 replicates), and then transfected with JFH1-RLuc subgenomic replicon RNA (provided by C. Rice of The Rockefeller University, New York, NY) using DMRIE-C (Thermo Fisher Scientific). Cell lysates were collected after 48 h and measured for *Renilla* luciferase activities per manufacturer’s instructions (Promega) using a POLARstar multidetection microplate reader (BMG Labtech).

#### HCV IRES-Mediated Translation Assay

Huh7.5.1 cells were transfected with various miRNA mimics as described above and incubated for 72 h. Cell were then transfected with pHCV-CLX-CMV RNA (encoding HCV IRES that directs the translation of a firefly luciferase reporter gene, provided by M. Niepmann of Giessen University, Giessen, Germany). After 24 h, cell lysates were obtained and firefly luciferase activity was subsequently measured per manufacturer’s instructions (Promega) using a POLARstar multidetection microplate reader (BMG Labtech).

#### HCV Single-Cycle Infection Assay

The core-defective, assembly deficient single-cycle infectious HCV (HCVsc) was generated from a trans-packaging system as previously described (Masaki *et al*. 2010; Li *et al*. 2014). HCVsc is able to enter and replicate viral RNA in hepatocytes but unable to produce progeny viruses, thus recapitulating only the early stages of the HCV life cycle (entry through genome replication), but not the late stages (virion assembly and secretion). In the assay, Huh7.5.1 cells were treated with various miRNA mimics at 25 nmol/L for 72 h in 96-well white plates (in 5 replicates) before infection with HCVsc. At 48 h post-infection, cells were harvested and measured for firefly luciferase activities per manufacturer’s instructions (Promega) using a POLARstar multidetection microplate reader (BMG Labtech).

#### HCV P7-Luc Infection

Huh7.5.1 cells grown in 96-well white microplates (in 5 replicates) were transfected with various miRNA mimics (at 25 nmol/L) or SMARTpool siRNAs (at 50 nmol/L). After 72 h, cells were infected with HCV P7-Luc (an HCVcc system encoding a *Renilla* luciferase reporter, provided by C. Rice of The Rockefeller University, New York, NY). At 48 h post-infection, cell lysates were prepared and analyzed for *Renilla* luciferase activities per manufacturer’s instructions (Promega) using a POLARstar multidetection microplate reader (BMG Labtech).

#### HCV Core Staining “Part-One” (Early Steps) and “Part-Two” (Late Steps)

For “part-one” assay, Huh7.5.1 cells were transfected with miRNA mimic control (Ctrl), miR-122 mimic or miR-135a mimic at a concentration of 25 nmol/L. After 72 h, cells were infected with the HCV JFH-1 strain. At 48 h post-infection, cells were fixed and immunostained for HCV core protein expression, using a purified anti-core monoclonal antibody (produced from 6G7 hybridoma cells). Cell nuclei were stained with Hoechst 33,342 (Invitrogen) at 1:5000 in PBS. Supernatants from “part-one” wells were transferred to infected naïve Huh7.5.1 cells in a new assay plate, starting “part-two”, which underwent the same procedures as “part-one”. “Part-one” and “part-two” HCV core staining recapitulate the early (entry to viral RNA translation and replication) and the late (virion assembly and secretion) stages of HCV infection, respectively (Li *et al*. 2009, 2014).

### Viral RNA Isolation and Quantification

Intracellular RNA was extracted from whole-cell lysates using the RNeasy Mini Kit (Qiagen). Viral RNA from supernatants (extracellular HCV RNA) was isolated using a QIAamp Viral RNA Mini Kit (Qiagen). Copy numbers of intracellular and extracellular HCV RNA were determined by Q-PCR using Verso 1-Step RT-qPCR Kit (Thermo Fisher Scientific) with previously described probe, primers, and parameters (Li *et al*. 2009). The relative amount of HCV RNA was normalized to the housekeeping control gene human 18S rRNA (Thermo Fisher Scientific). Q-PCR was performed on a ViiA 7 Real-Time PCR System (Applied Biosystems).

### 3′ UTR Assay

The LightSwitch 3′ UTR reporter GoClone constructs of various putative miR-135a targets were purchased from SwitchGear Genomics (Active Motif). Huh7.5.1 cells were transfected with miR-135a WT or MUT or with mimic control in 96-well white plates (in 5 replicates). After 24 h, cells were further transfected with 50 ng of GoClone 3′ UTR reporter plasmid using FuGENE 6 transfection reagent (Roche). Two days later, cells were lysed and total luciferase outputs were quantified using LightSwitch Luciferase Assay Reagent, according to the manufacturer’s instructions (SwitchGear Genomics) using a POLARstar multidetection microplate reader (BMG Labtech).

### Gene Expression Assay

Huh7.5.1 cells were transfected with various miRNA mimics (at 25 nmol/L) or SMARTpool siRNAs (at 50 nmol/L) for 72 h and then harvested. Total cellular RNA was extracted using an RNeasy Mini Kit (Qiagen). RNA quality and quantity were assessed on a Nanodrop spectrophotometer. cDNA was synthesized from total cellular RNA using a First Strand cDNA Synthesis Kit (Roche). The mRNA levels of target genes were subsequently determined by Q-PCR using gene-specific primers and probes (IDT) and FastStart Universal Probe Master (Roche) on an ABI ViiA 7 Real-Time PCR System. Relative mRNA levels were calculated using the ΔΔ*CT* method, with 18S rRNA (Applied Biosystems) as the internal control for normalization.

### MicroRNA Quantitative Real-Time PCR (Q-PCR) Assay

Huh7.5.1 cells or cells infected with HCV were vortexed to lyse. Human liver biopsies were lysed in TissueLyser LT bead mill (Qiagen). Total miRNA was isolated using a miRNeasy Mini Kit per manufacturer’s instructions (Qiagen). RNA was reverse transcribed using the TaqMan MicroRNA Reverse Transcription Kit (Applied Biosystems) according to the manufacturer’s instructions. miR-135a expression levels were determined by Q-PCR using TaqMan Universal PCR Master Mix (Applied Biosystems) and specific miRNA primers and probes (TaqMan MicroRNA Assays, Applied Biosystems). U6 snRNA was used as an internal control.

### Western Blotting

Huh7.5.1 cells were transfected with mimic control or miR-135a mimic. Cell lysates were obtained in RIPA buffer (Sigma) with complete protease inhibitor cocktail (Roche). Lysates were incubated on ice for 30 min, and then centrifuged at 17,500 ×g for 20 min at 4 °C. Supernatants were harvested and used for western blot analysis, on a ProteinSimple’s Wes instrument, per manufacturer’s instructions. 5 µL of diluted antibody per sample was applied. Multiple antibodies used for Western blotting were obtained commercially: MYD88 rabbit polyclonal antibody (1:500) (ab2064; Abcam), RIPK2 rabbit monoclonal antibody (1:500) (Clone D10B11; Cell Signaling), β-Tubulin monoclonal antibody (1:2000) (TUB 2.1; Sigma).

### ATPlite Assay

Huh7.5.1 cells were seeded in 96-well white assay plates at a density of 10,000 cells per well, and then treated with various miRNA mimics or miR-122 hairpin inhibitor at a final concentration of 25 nmol/L (in 5 replicates). After 72 h, cells were harvested and lysed with 50 µL of mammalian cell lysis solution (PerkinElmer). After 5 min incubation, 50 µL of ATPlite substrate solution (PerkinElmer) were added, and the luminescence in each well was subsequently measured using a POLARstar Omega multidetection microplate reader.

### Statistical Analysis

Results are presented as the means ± SE (standard errors). The two-tailed unpaired Student’s *t* test was used for statistical analysis. The level of significance is denoted in each figure (*P < 0.05, **P < 0.01. NS, not significant).

## Results

### miR-135a Transfection Enhances HCV Infection in Hepatocytes

**Fig. 1:**
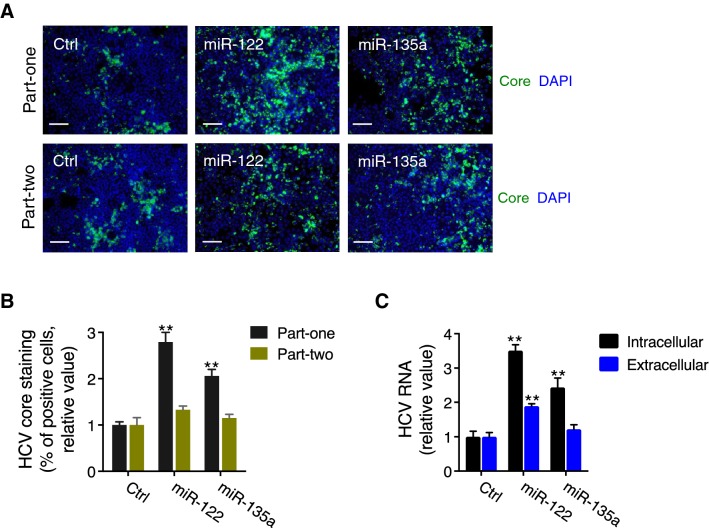
miR-135a transfection enhances HCV infection in hepatocytes. **A**, **B** Representative images (**A**) and quantitative analyses (**B**) of HCV core protein immunostaining in Huh7.5.1 cells transfected with miRNA mimic control (Ctrl), miR-122 mimic or miR-135a mimic, for “part-one” and “part-two” assays. Transfection of either miR-122 or miR-135a mimic exerted a greater effect on the “part-one” core staining assay that recapitulates viral entry, translation or genome replication, than the “part-two” assay, which recapitulates the later steps of the HCV life cycle. Green, HCV core; blue, nuclei. Scale bars, 100 μm. **C** Quantification of intracellular and extracellular HCV RNA levels after miR-135a or miR-122 mimic overexpression in Huh7.5.1 cells. **B**, **C** Values are normalized relative to Ctrl (as 1), and error bars represent the standard error of the mean (SEM), n = 3. **P < 0.01, determined by Student’s *t* test.

To validate the effect of miR-135a on HCV infection identified from the functional genomics screen (Li *et al*. 2017), we performed multiple virological assays using synthetic miR-135a mimic and HCV cell culture system (HCVcc). Huh7.5.1 cells were treated with miR-135a mimic for three days before infection with HCV JFH-1 strain. The two-part HCVcc infection assays examining HCV core protein expression by immunostaining were conducted as described previously (Li *et al*. 2009, 2014). To summarize, the “part-one” assay detects an effect on the early stages of HCV life cycle, from entry to viral RNA translation and replication, whereas the “part-two” assay defines the impact on the later stages of viral infection, including assembly and release. Transfection of hepatocytes with the miR-135a mimic significantly enhanced HCV core protein expression in the “part-one” infection assay (Fig. 1A, 1B). miR-135a overexpression also drastically elevated intracellular HCV RNA levels (Fig. 1C). These effects of miR-135a were comparable to those observed following miR-122 mimic transfection of Huh7.5.1 cells, suggesting that both miRNAs may play similar roles in modulating HCV infection.

### miR-135a Preferentially Regulates HCV Genome Replication

**Fig. 2:**
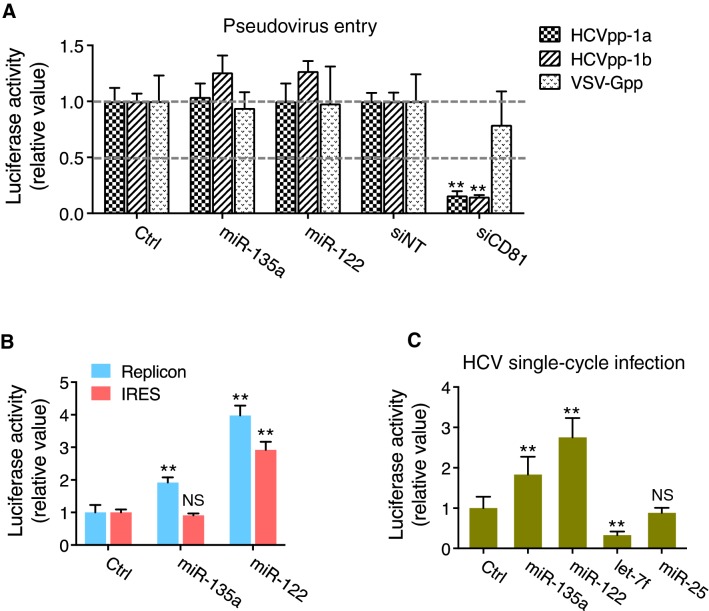
Impacts of miR-135a on the HCV life cycle. **A** miR-135a or miR-122 mimic transfection has no effect on entry of luciferase-encoding pseudotyped viruses bearing HCV (HCVpp, genotypes 1a and 1b) or VSV (VSV-Gpp, serving as a control) envelope proteins in Huh7.5.1 cells. siRNA-mediated silencing of CD81, a major HCV entry factor, was used as a positive control. **B** Effects of miR-135a or miR-122 on HCV RNA replication and IRES-mediated translation, assessed by HCV subgenomic replicon and IRES assays. **C** Effects of various indicated miRNA mimics on HCV single-cycle infection assay (HCVsc). Previously identified early-stage proviral (miR-122) and antiviral (let-7f), and late-stage antiviral (miR-25) miRNAs were included as positive (miR-122 and let-7f) or negative (miR-25) controls. Values are normalized relative to the mimic control (set as 1), and error bars represent the standard error of the mean (SEM), n = 5. **P < 0.01 determined by Student’s *t* test. NS, not significant.

We next examined the exact steps of the HCV life cycle that are influenced by miR-135a expression. First, we conducted viral entry assays using HCV pseudoparticles (HCVpp) of genotypes 1a and 1b, and control pseudovirus VSV-Gpp that bears the vesicular stomatitis virus glycoprotein. Transfection of miR-135a or miR-122 mimic in Huh7.5.1 cells did not exert noticeable effects on HCVpp or VSV-Gpp entry, which was in contrast to the strong inhibitory effect executed by small interfering RNA (siRNA)-mediated knockdown of CD81, a major HCV receptor (Fig. 2A), suggesting that miR-135a and miR-122 are not involved in HCV entry. miR-135a overexpression nevertheless significantly increased HCV replicon activity in the subgenomic replicon assay (Fig. 2B). Unlike miR-122, which affects both HCV internal ribosomal entry site (IRES)-mediated translation and viral RNA replication, miR-135a transfection had no effect on HCV RNA translation (Fig. 2B). These data indicate that miR-135a specifically acts on the viral genome replication step to promote HCV infection. Moreover, miR-135a transfection in Huh7.5.1 cells increased the infection of single-round infectious HCV (HCVsc) (Fig. 2C). HCV single-cycle infection assay using HCVsc recapitulates viral entry, translation and replication, but not assembly or secretion of the virions (Li *et al*. 2014), thus confirming the function of miR-135a in modulating an early step of the HCV life cycle.

### The Mutant Form of miR-135a Lacks the miRNA’s Proviral Effect

**Fig. 3:**
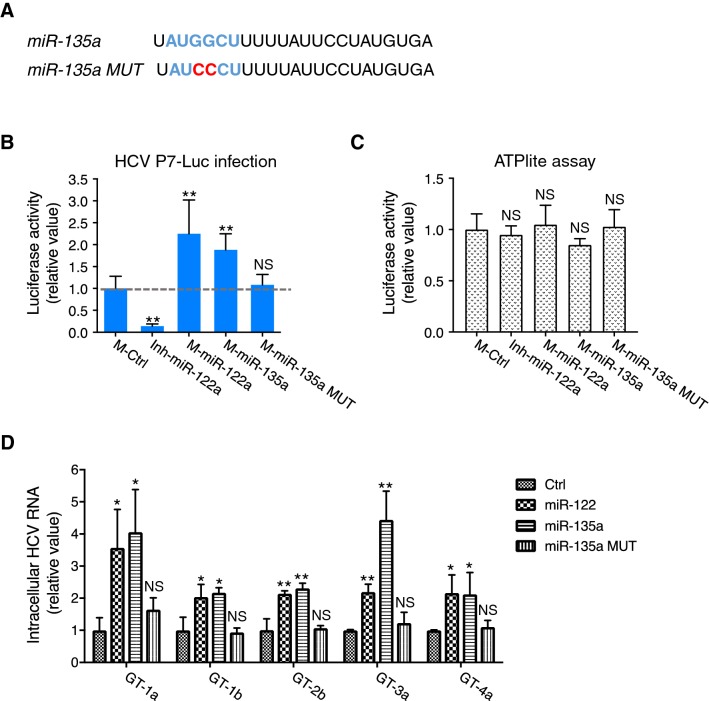
miR-135a seed sequence mutant form abrogated miR-135a’s proviral function. **A** RNA sequences of miR-135a wildtype (WT) and mutant (MUT) forms. The seed sequence is featured in blue, and the two point mutations on miR-135a MUT are shown in red. **B** Effects of WT or MUT miR-135a on HCVcc-Luc infection. miR-122 mimic (proviral) or hairpin inhibitor (antiviral) were used as positive controls. Relative luciferase activities upon treatment of various miRNAs are shown. **C** Huh7.5.1 cells were treated with each indicated miRNA, and ATPlite assays that examine viability of the cells and potential cytotoxicity of the miRNAs were subsequently conducted. **B**, **C** M, miRNA mimic; Inh, hairpin inhibitor. **D** Quantification of intracellular HCV RNA levels in miR-122, miR-135a WT or miR-135a MUT– treated Huh7.5.1 cells infected with HCV of various major genotypes. miR-135a, like miR-122, is a pan-proviral cellular factor for HCV. **B**, **C**, **D** Values are normalized relative to M-Ctrl (as 1), and error bars represent the standard error of the mean (SEM), n = 5 (**B**, **C**) or 3 (**D**). *P < 0.05, **P < 0.01 determined by Student’s *t* test. NS, not significant.

MicroRNA-mediated gene regulation is achieved through partial pairing between the target mRNA and six to seven nucleotides at the 5′end of the miRNA, called the seed sequence (Bartel 2009). To investigate whether direct miRNA–mRNA binding is responsible for miR-135a’s effect on HCV RNA replication, we conducted mutagenesis assay. We synthesized a mutant miR-135a that contains guanine (G) to cytosine (C) mutations at the 3^rd^ and 4^th^ positions of its seed sequence (miR-135a MUT) (Fig. 3A) and conducted P7-Luc HCV infection assay. miR-135a mimic transfection significantly enhanced HCV-Luc activity in Huh7.5.1 cells; whereas this proviral phenotype was absent in cells transfected with miR-135a MUT (Fig. 3B). As expected, miR-122 mimic or hairpin inhibitor considerably enhanced or reduced HCV infection, respectively (Fig. 3B). Transfection of any of these miRNAs was not associated with apparent cytotoxicity (Fig. 3C).

### Pan-Genotypic Effect of miR-135a on HCV Infection

In addition to modulating the replication of genotype 2a HCV as described above, we also showed that overexpression of miR-135a in Huh7.5.1 cells significantly enhanced the infection of other major HCV genotypes (including 1a, 1b, 2b, 3a and 4a) (Fig. 3D). Transfection of miR-135a MUT failed to achieve the miR-135a’s proviral effect on infection of various genotypes (Fig. 3D). Hence, miR-135a modulates HCV infection in a pan-genotypic manner that depends on the binding of its seed sequence to target mRNAs.

### miR-135a Directly Targets Multiple HCV Restriction Factors in Hepatocytes

**Fig. 4:**
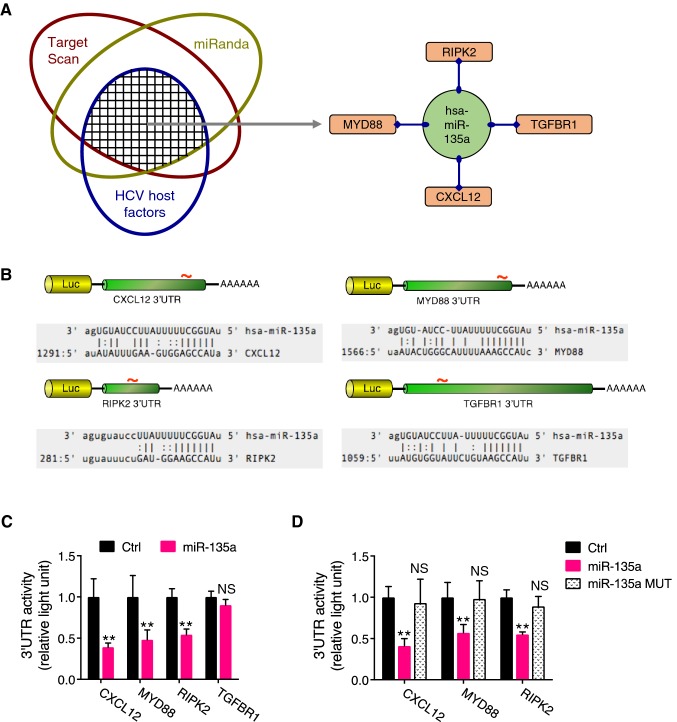
Identification of the *bona fide* miR-135a targets among HCV host dependencies. **A** Schematics for systematic identification of putative miR-135a targets. Bioinformatics-based target prediction, in line with assessing phenotypic effects on HCV infection, derived four antiviral host factors as phenotypic-specific miR-135a candidate targets (in orange rounded rectangles) for further validation. **B** Luciferase reporter-harboring 3′ UTR constructs of various putative miR-135a targets. Each 3′ UTR encodes one miR-135a seed matching site. **C** miR-135a mimic transfection diminished 3′ UTR activities of CXCL12, MYD88 and RIPK2, but not that of TGFBR1. **D** miR-135a MUT transfection annulled the regulatory effects on CXCL12, MYD88 and RIPK2 3′ UTR activities. **C**, **D** All values are normalized relative to Ctrl (as 1), and error bars represent the standard error of the mean (SEM), n = 5. **P < 0.01 determined by Student’s *t* test. NS, not significant.

MicroRNAs predominately exert their effects by targeting the 3′ UTR of an mRNA transcript, leading to a decrease in protein expression of the target (Guo *et al*. 2010). To explore the cellular targets that mediate miR-135a’s proviral functions, we applied a combined functional and bioinformatics-derived analysis that was employed for the discovery of *bona fide* targets for various HCV-relevant miRNAs in our previous functional genomics study (Li *et al*. 2017). Specifically, two major miRNA target prediction tools, TargetScan and miRanda-mirSVR, were used to predict biological targets of miR-135a. These putative targets were then cross-referenced with previously identified and validated HCV host dependencies through our genome-wide screens (Li *et al*. 2009, 2014) and literature datamining (Supplementary Table S1). Four HCV restriction factors, C-X-C motif chemokine ligand 12 (CXCL12), myeloid differentiation primary response 88 (MyD88), receptor-interacting serine-threonine kinase 2 (RIPK2), and TGF-β receptor 1 (TGFBR1), were thus defined as putative phenotype-specific miR-135a targets (Fig. 4A). These four proteins have been shown to inhibit HCV infection at the viral genome replication stage (Li *et al*. 2014). Therefore, if they were targeted and repressed by miR-135a, HCV replication would increase.

Each of these putative targets possesses a miR-135a seed sequence match site in its 3′ UTR (Fig. 4B). To evaluate whether miR-135a truly binds to these genes, we performed 3′ UTR assays. The entire 3′ UTR of the gene of interest was inserted downstream of a luciferase reporter gene (Fig. 4B). The luciferase activity was then measured after co-transfection of these 3′ UTR constructs with either the miR-135a mimic or a non-targeting control. miR-135a overexpression significantly reduced the luciferase activities of CXCL12, MYD88 and RIPK2 3′ UTR constructs, but not that of the TGFBR1 3′ UTR (Fig. 4C), suggesting regulation of the former three 3′ UTRs by miR-135a. The function of miR-135a in regulating CXCL12, MYD88 and RIPK2 3′ UTR activities was further confirmed by testing the miR-135a MUT, which did not inhibit CXCL12, MYD88 or RIPK2 3′ UTR activity, due to a loss of seed-sequence binding to these targets (Fig. 4D).

### miR-135a Downregulates CXCL12, MYD88 and RIPK2 Hepatocellular Expression to Enhance HCV Replication

**Fig. 5:**
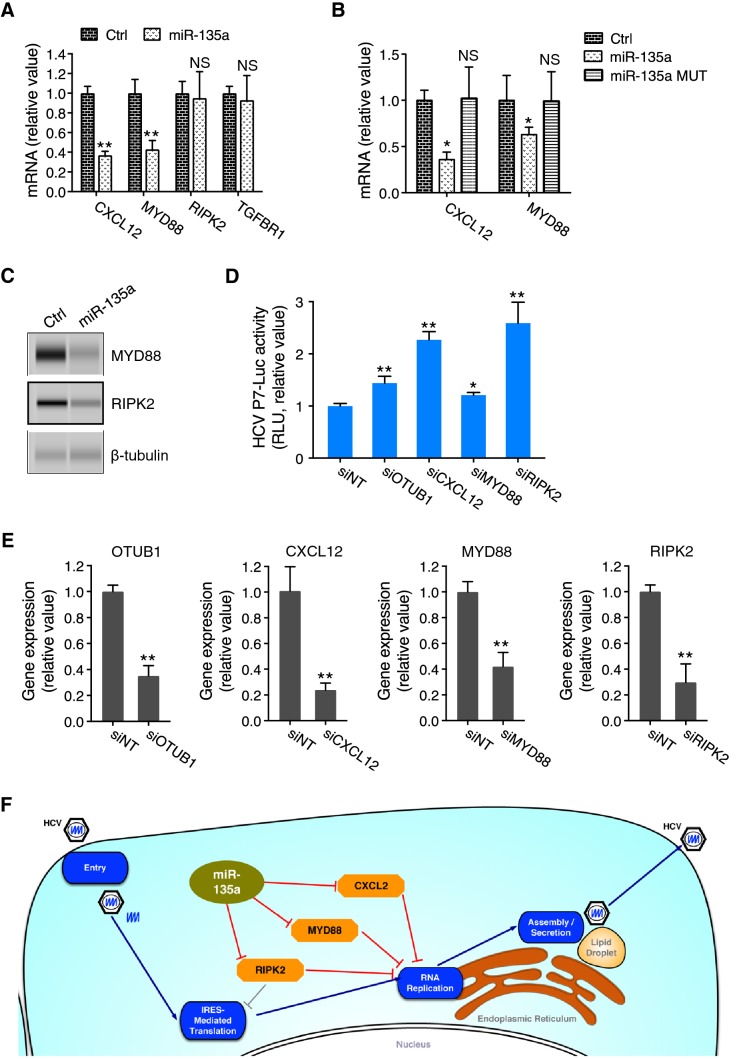
miR-135a directly targets and represses the expression of the antiviral host factors CXCL12, MYD88, and RIPK2 to promote HCV genome replication. **A** Transfection of miR-135a mimic in Huh7.5.1 cells abated mRNA levels of CXCL12 and MYD88, but not that of RIPK2 nor TGFBR1. **B** miR-135a MUT overexpression abrogated the regulatory effects of miR-135a on CXCL12 and MYD88 mRNA levels. **C** Effects of miR-135a mimic transfection on protein levels of MYD88 and RIPK2, determined by Western blot. β-tubulin was used as a loading control. **D** Depletion of various indicated host factors by siRNA treatment enhanced HCVcc-Luc infection in Huh7.5.1 cells. siRNA against OTUB1 (siOTUB1), a previously identified host restriction factor for HCV (Li *et al*., 2014), was used as a positive control. **E** Knockdown efficiencies of the above-used siRNAs. OTUB1, CXCL12, MYD88 and RIPK2 mRNA levels were determined by quantitative RT-PCR (Q-PCR). **F** Schematic map illustrating that miR-135a (in light green oval) directly targets antiviral factors CXCL12, MYD88, and RIPK2 (in orange octagons) to exert its function in enhancing HCV RNA replication. **A**, **B**, **D**, **E** All values are normalized relative to Ctrl or siNT (as 1), and error bars represent the standard error of the mean (SEM), n = 3 (**A**, **B**, **E**) or 5 (**D**). *P < 0.05, **P < 0.01 determined by Student’s *t* test. NS, not significant.

MicroRNAs are known to repress target gene expression primarily through mRNA destabilization and degradation (Bartel 2009; Guo *et al*. 2010). As such, we measured the mRNA levels of four putative miR-135a targets via Q-PCR in miR-135a-overexpressed hepatocytes. miR-135a mimic transfection markedly decreased the mRNA levels of CXCL12 and MYD88 (Fig. 5A), while its mutant form did not exert any regulatory effect (Fig. 5B), suggesting both mRNAs are directly manipulated by miR-135a.

For RIPK2, while its 3′ UTR was subject to miR-135a regulation (Fig. 4C, 4D), its mRNA expression level did not show a decrease after miR-135a transfection (Fig. 5A). We then assessed its protein level and showed that miR-135a mimic overexpression substantially decreased RIPK2 protein level in Huh7.5.1 cells (Fig. 5C). Similarly, the protein level of an above confirmed miR-135a target–MYD88–was also considerably reduced in cells transfected with miR-135a (Fig. 5C). These data are in line with the notion that although lowered mRNA levels account for the major form of miRNA-mediated modulation, a few “outliers” in which translation is suppressed without noticeable mRNA degradation exist, such as in the case of RIPK2. We further confirmed the antiviral effects of CXCL12, MYD88 and RIPK2 that mediate miR-135a’s proviral functions. Knockdown of these host genes in Huh7.5.1 cells by siRNA treatment significantly enhanced P7-Luc HCV infection (Fig. 5D, 5E).

Collectively, the data generated a schematic map that illustrates miR-135a’s proviral function and mechanism of action. We demonstrated that miR-135a selectively targets and inhibits the expression of three critical HCV restriction factors, CXCL12, MYD88 and RIPK2, to specifically promote viral genome replication (Fig. 5F).

### HCV Infection Induces miR-135a Expression in Hepatocytes and Human Liver

**Fig. 6:**
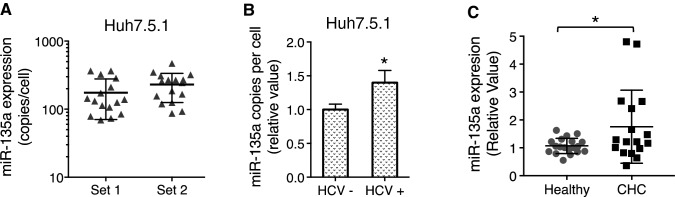
HCV infection upregulates hepatic expression of miR-135a. **A** Copies of miR-135a transcripts per cell in Huh7.5.1 cells (two sets), examined by Q-PCR gene expression assays. Each dot represents one hepatocyte that was measured. **B** HCV infection induces miR-135a expression in Huh7.5.1 cells. **C** Hepatic abundance of miR-135a is significantly higher in the livers of chronic hepatitis C (CHC) patients compared to those of healthy controls, quantified by Q-PCR. Each dot represents one individual’s liver tissue.

We next showed that miR-135a is expressed in human hepatocytes. Q-PCR-based miRNA quantification assay demonstrated an average of 100 ~ 300 copies of transcripts per cell in cultured Huh7.5.1 cells (Fig. 6A). HCV infection significantly increased the abundance of miR-135a in these hepatocytes (Fig. 6B). Furthermore, in liver biopsies of chronic hepatitis C (CHC) patients, miR-135a expression levels are significantly higher than those of healthy controls (Fig. 6C).

## Discussion

Cellular miRNAs have been shown to play important regulatory roles in HCV infection and virus–host interactions (Singaravelu *et al*. 2014). Indeed, both the HCV genome and viral host dependencies can be targeted by cellular miRNAs, which either positively or negatively regulate productive HCV infection (Sarnow *et al*. 2006; Singaravelu *et al*. 2014). HCV may also impact the expression landscape of host miRNAs and co-opt their functions to establish viral persistence and induce various pathological conditions in the liver (Schwerk *et al*. 2015). The exact roles and underlying mechanisms of cellular miRNAs in modulating HCV infection and associated liver diseases, nevertheless, remain to be elucidated.

Recently, applying integrative functional genomics and systems biology approaches, we globally dissected HCV–cellular miRNA interactions. We conducted a combined genome-wide miRNA mimic and hairpin inhibitor phenotypic screen, followed by miRNA–mRNA transcriptomics analyses, and identified both proviral and antiviral miRNAs that physiologically interact with HCV. These miRNAs were then functionally linked to particular steps of HCV life cycle and related viral host dependencies, thereby revealing extensive cellular miRNA–mRNA regulatory networks associated with HCV infection and propagation (Li *et al*. 2017). Follow-up studies towards several of the most significant and relevant miRNA hits concerning their modes of actions in modulating HCV-mediated liver pathogenesis have been carried out. These include miR-135a, a proviral cellular miRNA identified from the mimic screen (Li *et al*. 2017).

In the present study, we elucidated the precise function and the mode of action of miR-135a in modulating HCV infection. Through a panel of virological assays, we identified that miR-135a preferentially induces the viral genome amplification but does not alter the other stages of the HCV life cycle. We determined that miR-135a enhances the abundance of HCV RNA in hepatocytes via its antagonism of cellular antiviral pathways, by targeting and repressing the expression of several critical host restriction factors, including CXCL2, MYD88 and IRPK2, at either the transcriptional or translational level. Consistent with the role of miR-135a, these antiviral factors were shown to restrict HCV RNA replication.

Among the validated miR-135a cellular targets, CXCL2 is an antimicrobial gene serving as a ligand for the G-protein couple receptor CCR4 that plays a role in diverse cellular functions, including immune surveillance, inflammatory response, tissue homeostasis, and tumor growth and metastasis (Murphy and Heusinkveld 2018). MYD88 is a key regulator of the innate and adaptive immune responses. In particular, it serves as an essential signal transducer in the interleukin-1 and Toll-like receptor signaling pathways, which govern the activation of versatile proinflammatory genes (Akira and Takeda 2004). The serine/threonine protein kinase RIPK2 is also an important component of signaling complexes in both the innate and adaptive immune pathways. Interestingly, in response to various stimuli including viral infections, RIPK2 potently activates NF-κB and induces apoptotic cell death as a host defense mechanism (McCarthy *et al*. 1998). Given their versatile pathophysiological functions, these genes may mediate not only miR-135’s proviral function, but also its putative effects on multiple hepatic disorders, particularly HCV-associated liver malignancy.

One such liver malignancy is hepatocellular carcinoma (HCC), which is the predominant form of liver cancer and overall the fifth most prevalent tumor type and the second leading cause of cancer-related deaths worldwide. In the United States, HCC is the fastest growing cause of cancer-related mortality. Chronic infection with HCV is the leading risk factor for HCC. HCV is unique among tumor viruses in that it does not encode oncoproteins nor there is integration of the viral genome into host chromosomal DNA. Thus, the virus initiates and promotes HCC development mainly through indirect effects by deregulating various cellular processes, including hepatocyte proliferation, steatosis, oxidative stress, inflammation, immune responses, and fibrosis/cirrhosis. The specific molecular mechanisms supporting HCV-mediated HCC initiation and progression, nevertheless, are poorly understood. To further explore these mechanisms we looked into miR-135a, the expression of which has been shown broadly induced across various cancer types, including HCC (Van Renne *et al*. 2018). Elevated miR-135a expression, on the other hand, promotes cancer cell invasion and thus contribute to metastasis and progression of HCC (Liu *et al*. 2012). The mechanisms by which miR-135a exerts these oncogenic effects remain unclear.

Accumulating evidence has demonstrated that miRNA machinery constitutes an important part of viral host dependencies, and host miRNAs may modulate viral infections either directly, through targeting viral genomes, or indirectly, through regulation of virus-associated cellular pathways (Sarnow *et al*. 2006). Viruses, such as Epstein–Barr virus (EBV), enterovirus, and eastern equine encephalitis virus, can benefit from the expression of certain miRNAs, and may further induce these miRNAs for productive infection and pathogenesis (Trobaugh *et al*. 2014). Endogenous cellular miRNAs can also exert significant antiviral effects on several human viruses, including vesicular stomatitis virus (VSV) and the retrovirus human immunodeficiency virus (HIV) (Lecellier *et al*. 2005). Nevertheless, many viruses seem to be resistant to suppression or augmentation by the host miRNome during their replication cycle (Bogerd *et al*. 2014). These viruses may have evolved strategies to impede endogenous miRNA functions or to avoid direct miRNA targeting (Cullen 2013). On the other hand, given the versatile regulatory roles of miRNAs in any given biological process (such as viral infection), multiple concurrent proviral or antiviral signals may co-exist and be tightly controlled by the miRNA machinery to maintain a balanced state.

HCV infection closely interacts with the miRNA machinery. Besides miR-122, and miR-135a characterized in this study, a number of other cellular miRNAs have been shown to be critical regulators of HCV infection. Among them, miR-196 may target Bach1, and hence up-regulate the antiviral factor HMOX1 to restrict HCV replication (Hou *et al*. 2010). miR-29, a cellular miRNA implicated in development of liver fibrosis (Roderburg *et al*. 2011), was demonstrated to block HCV propagation and its expression is down-regulated by HCV (Bandyopadhyay *et al*. 2011). In addition, miR-199a-3p, an HCC-suppressive miRNA (Hou *et al*. 2011), possesses a conserved binding site in the HCV 5′ UTR. And overexpression of this miRNA significantly inhibits HCV infection (Murakami *et al*. 2009). Besides miR-199a, several other miRNAs including miR-448 and miR-196, with putative target sequences on the viral genome, have been shown to attenuate HCV genome replication (Pedersen *et al*. 2007). However, direct functional interactions of these miRNAs with the viral genome have not been shown. Interestingly, some of these miRNAs seem to be induced by interferons, with inhibition of their activities impairing IFN’s antiviral effect against HCV (Pedersen *et al*. 2007). Hence, HCV may manipulate these IFN-activated miRNAs to limit replication to a level appropriate for persistent viral infection.

In summary, as major factors in controlling various cellular processes, miRNAs represent a compelling subject of investigation in the study of HCV infection and virus-mediated liver diseases. Understanding miRNA-associated mechanisms and major signaling pathways involved in HCC pathogenesis is instrumental for target-directed drug discovery to optimize the current treatment regimen for liver cancer.

## Electronic supplementary material

Below is the link to the electronic supplementary material.

Supplementary Table S1 HCV host dependencies cross miR-135a targets (XLSX 47 kb)
